# Supplementary material for: Antimicrobial activity and synergistic effect of phage-encoded antimicrobial peptides with colistin and outer membrane permeabilizing agents against Acinetobacter baumannii
Source: PeerJ. 2024 Dec 24;12:e18722. doi: 10.7717/peerj.18722 (PMC11674141; doi:10.7717/peerj.18722)

Figure S1 ***G. mellonella*** **infection assays of phage encoded peptide PE04-1 against** ***A. baumannii*** **ATCC 19606**

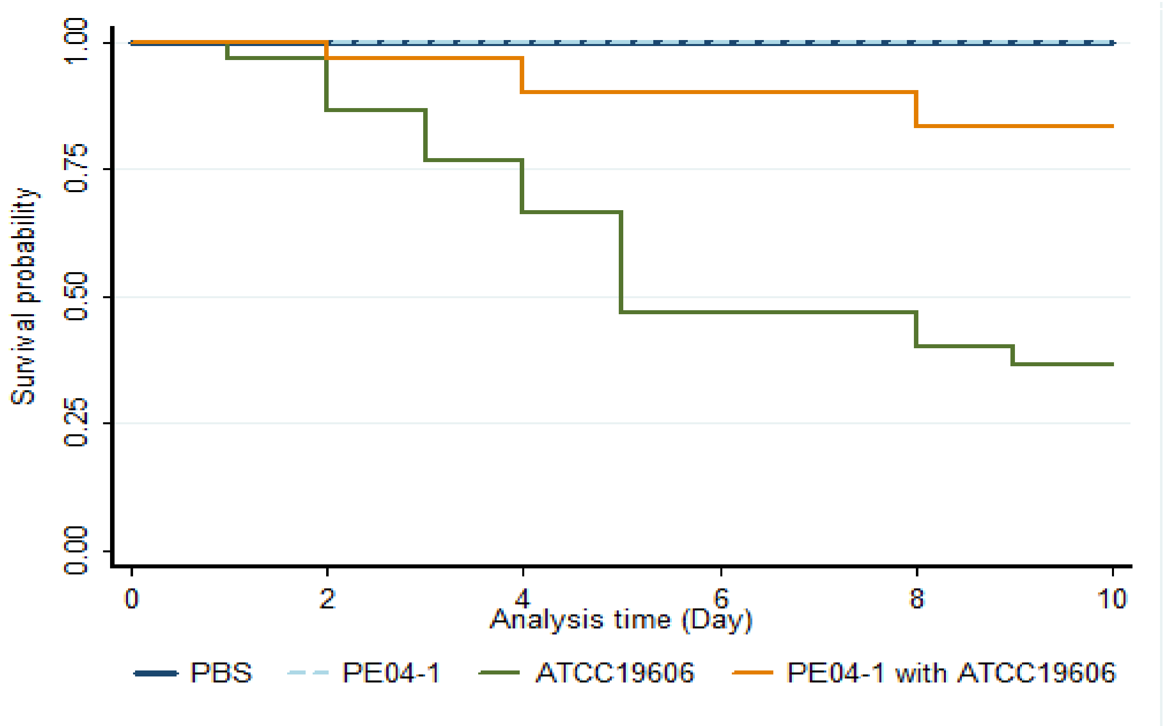

FigureS2 ***G. mellonella*** **infection assays of phage encoded peptide PE04-1 (NH_2) against** ***A. baumannii*** **ATCC 19606**

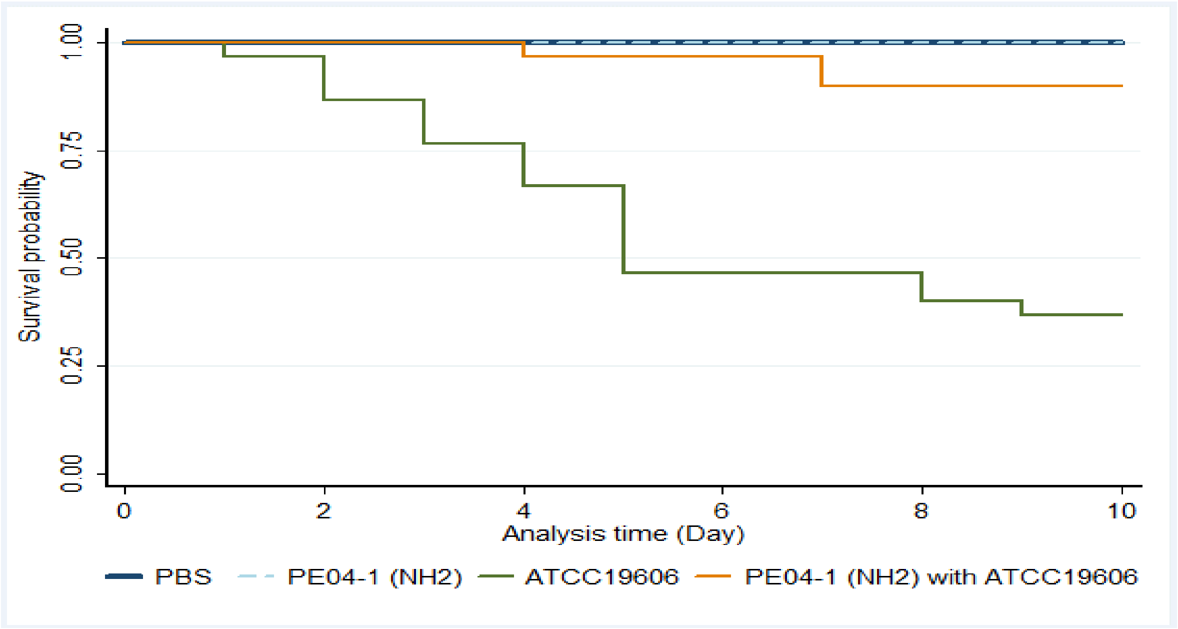

FigureS3 *G. mellonella* infection assays of phage encoded peptide PE04-2 against *A. baumannii* ATCC 19606

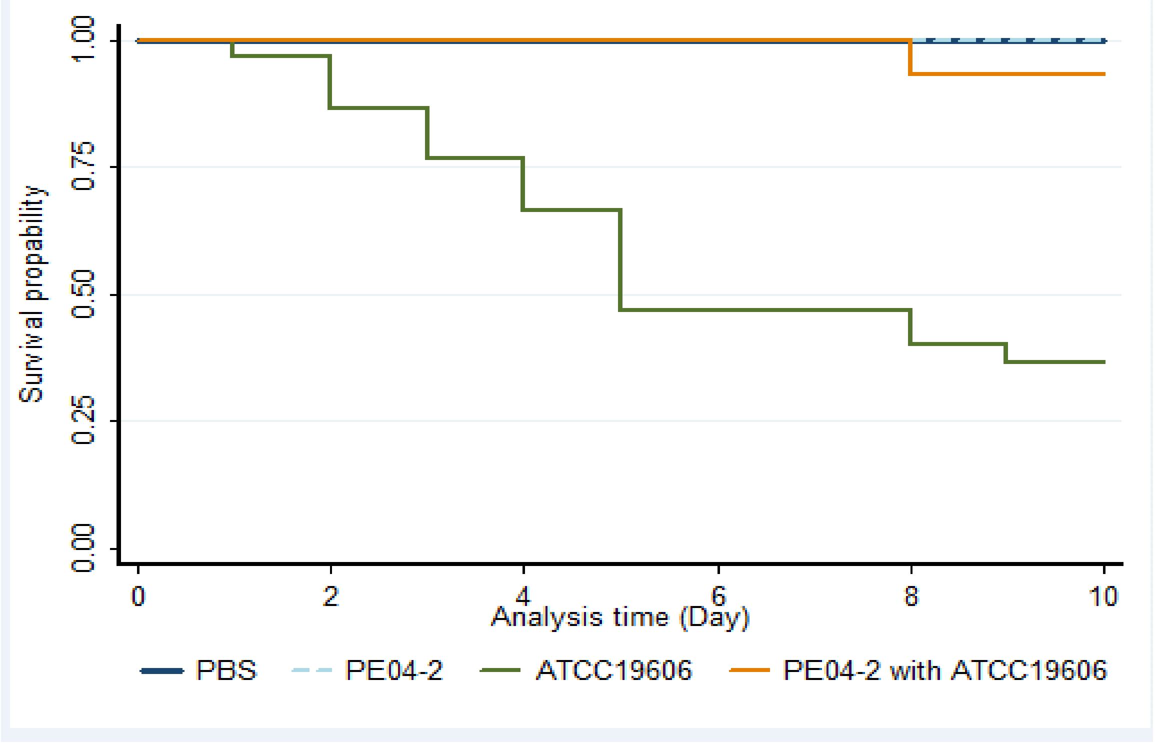

Supplement: Supplemental Information 1 [file peerj-12-18722-s001.pdf]
