# Supplementary material for: Antimicrobial activity and synergistic effect of phage-encoded antimicrobial peptides with colistin and outer membrane permeabilizing agents against Acinetobacter baumannii
Source: PeerJ. 2024 Dec 24;12:e18722. doi: 10.7717/peerj.18722 (PMC11674141; doi:10.7717/peerj.18722)
Supplement: Supplemental Information 7 [file peerj-12-18722-s007.pdf]

Group 1	Day 1	Day 5	Day 10
Not Injection	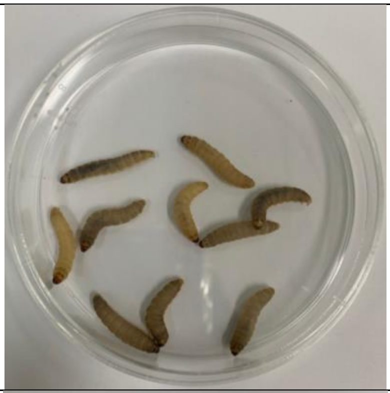	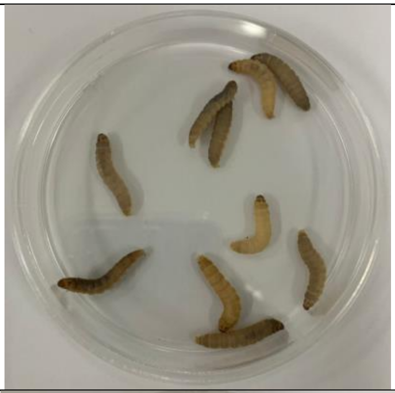	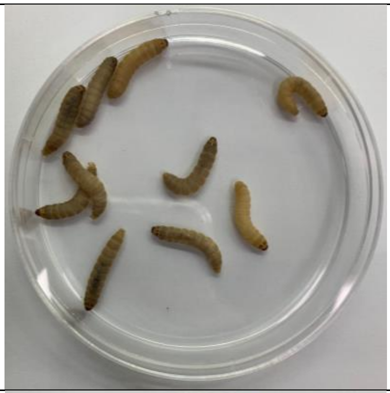
Only PBS	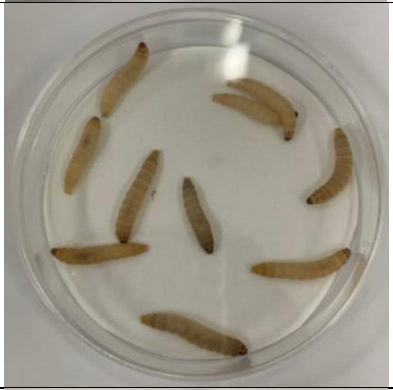	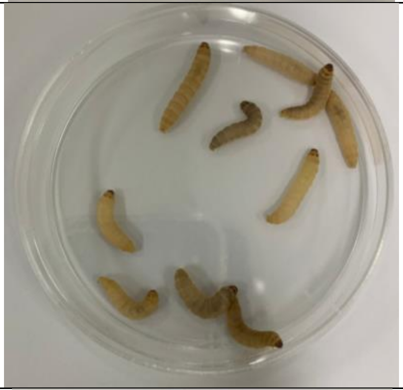	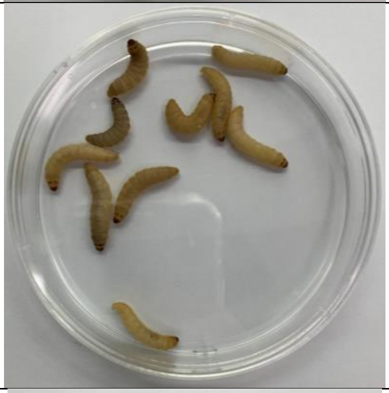
Only CR04-WT	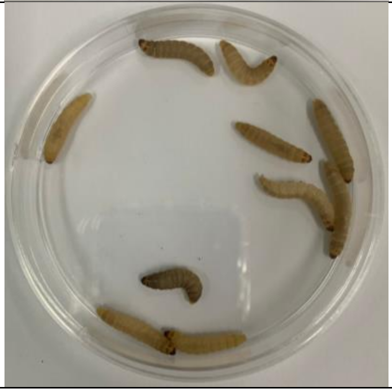	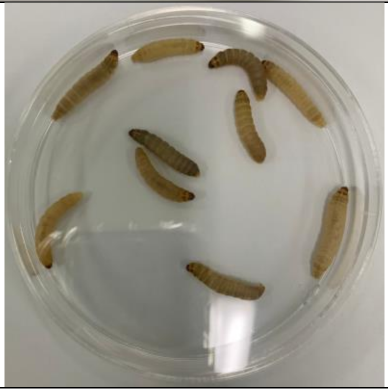	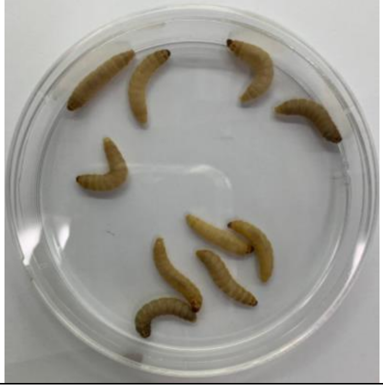

Only CR04-NH_2	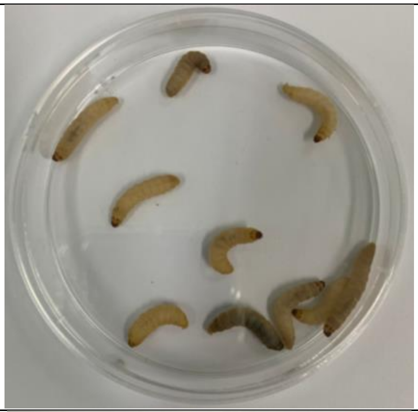	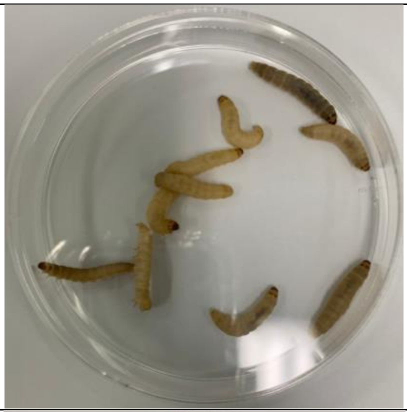	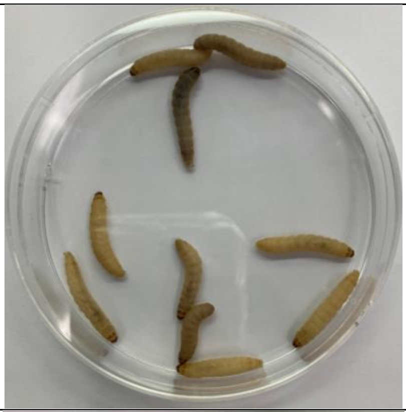
CR04-CPP	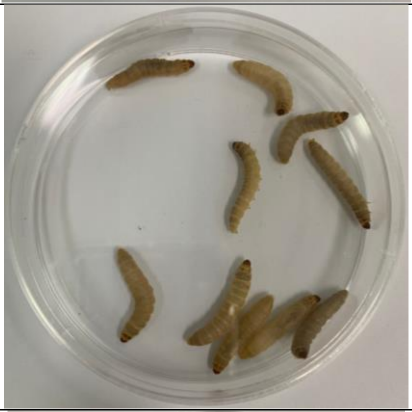	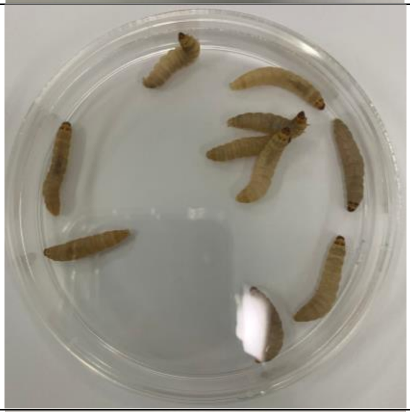	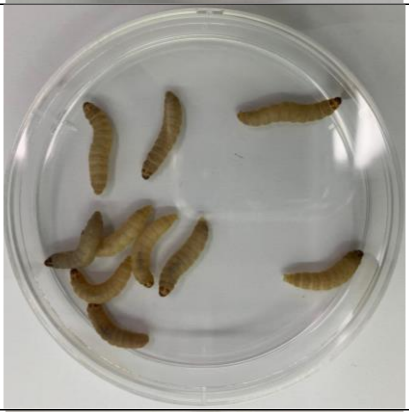
Only AB329	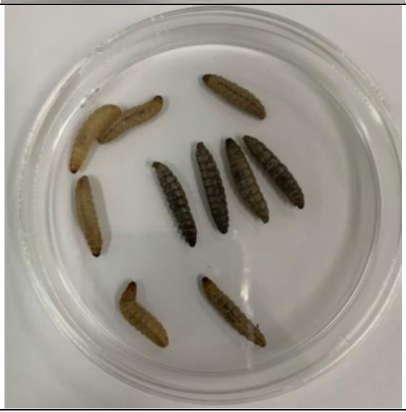	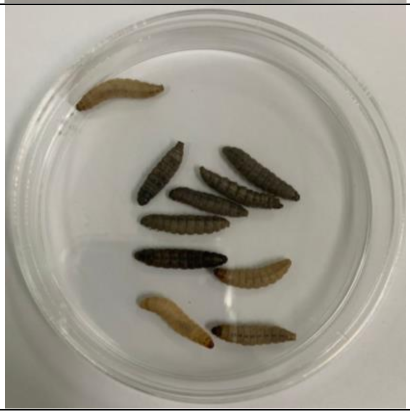	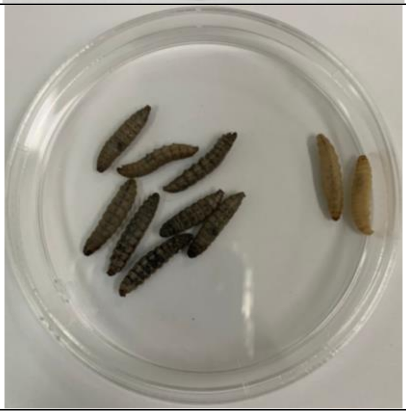

CR04-WT with AB329	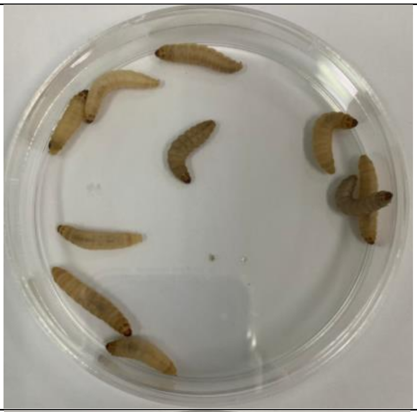	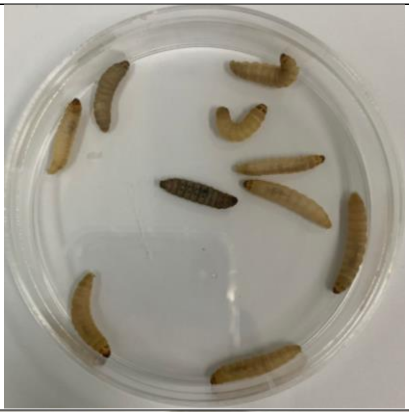	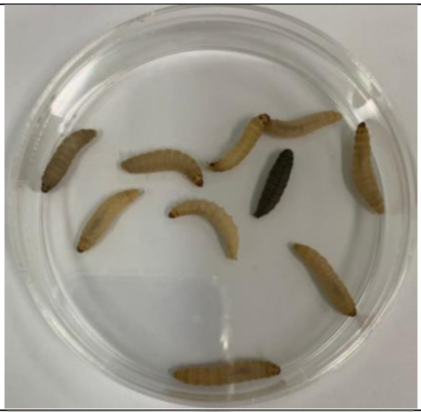
CR04-NH_2 with AB329	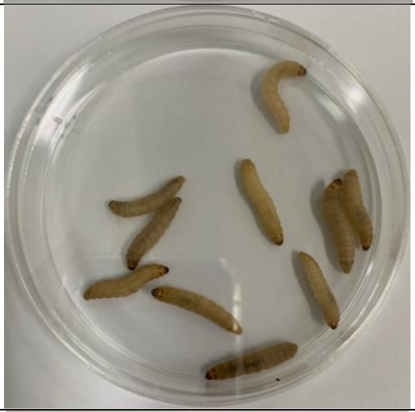	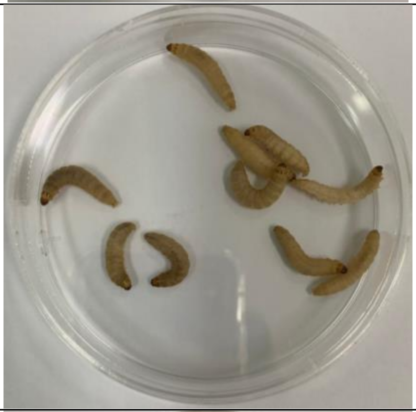	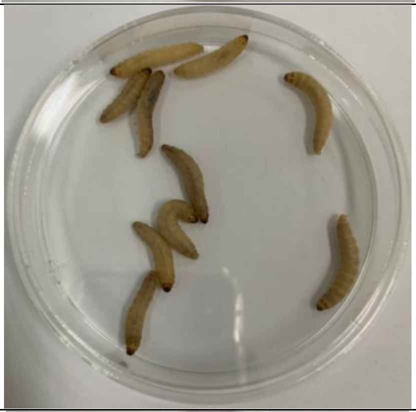
CR04-CPP with AB329	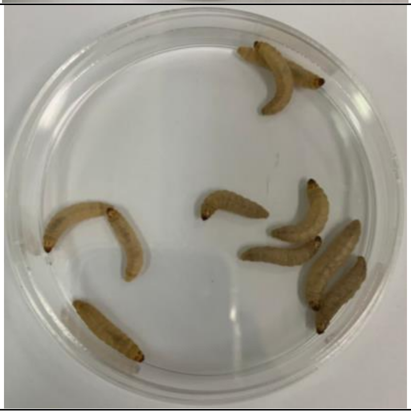	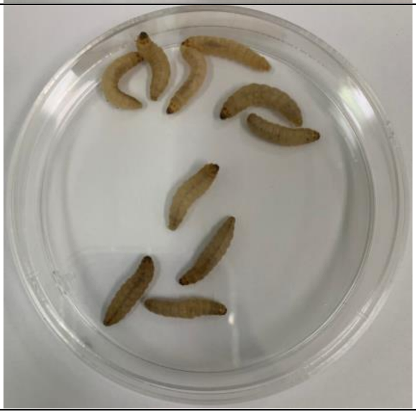	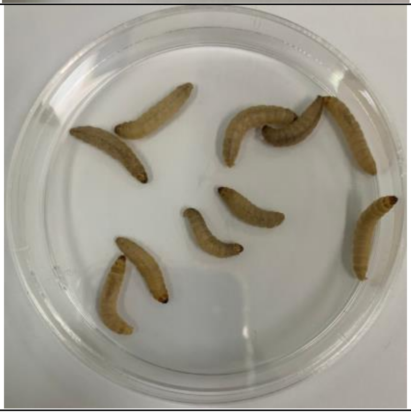

Post-Treatment CR04-WT with AB329	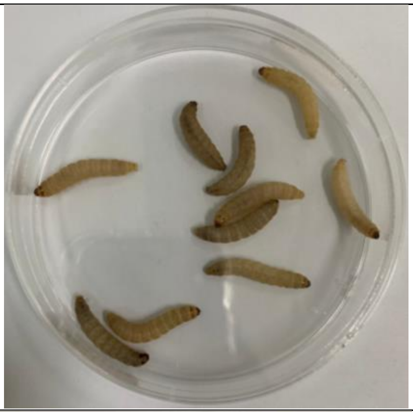	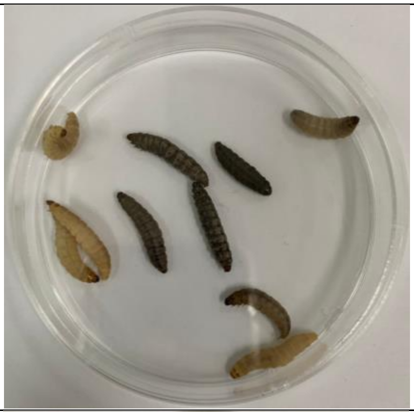	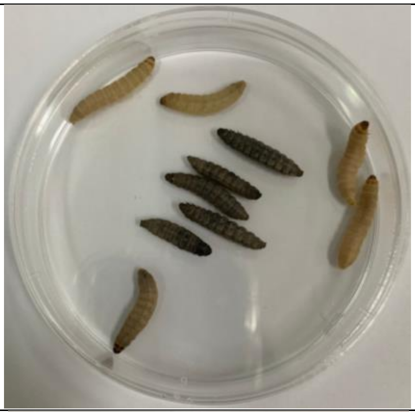
Post-Treatment CR04-NH_2 with AB329	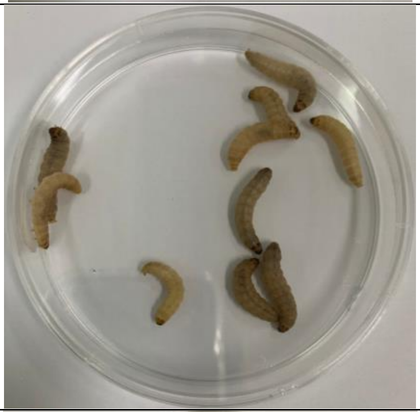	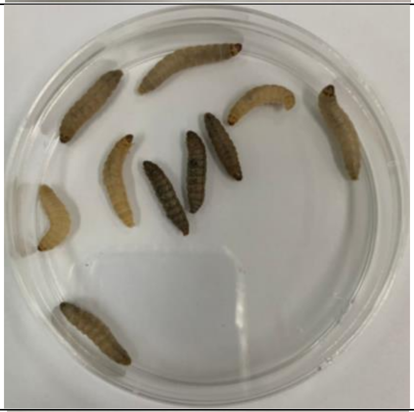	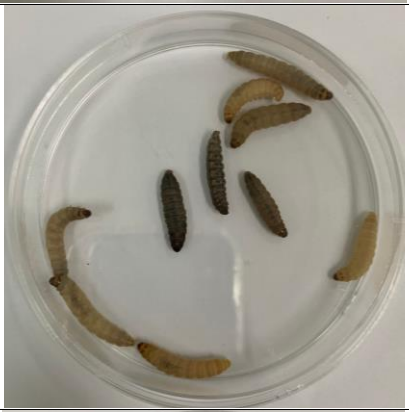
Post-Treatment CR04-CPP with AB329	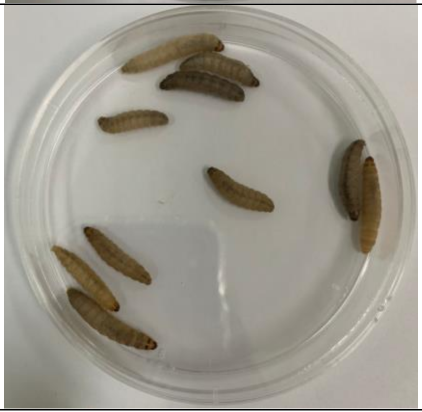	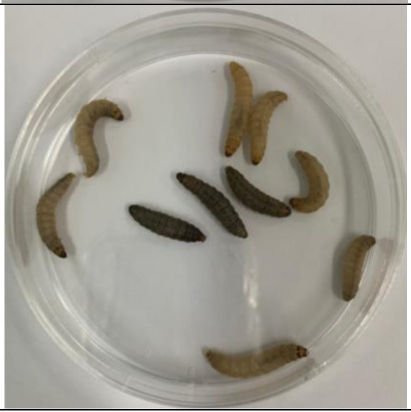	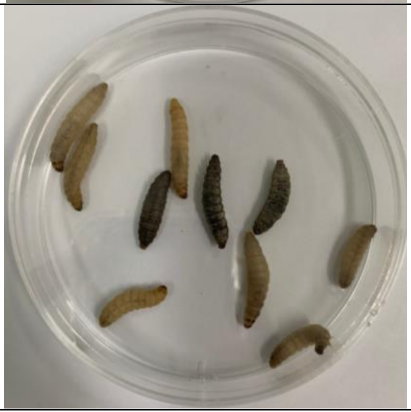
